# Ubiquitin Lys 63 chains – second-most abundant, but poorly understood in plants

**DOI:** 10.3389/fpls.2014.00015

**Published:** 2014-01-31

**Authors:** Konstantin Tomanov, Christian Luschnig, Andreas Bachmair

**Affiliations:** ^1^Max F. Perutz Laboratories, Department of Biochemistry and Cell Biology, Center for Molecular Biology, University of ViennaVienna, Austria; ^2^Department of Applied Genetics and Cell Biology, University of Natural Resources and Life SciencesVienna, Austria

**Keywords:** ubiquitin Lys63 chains, cell signaling, vacuolar sorting, endoctosis, iron response, DNA repair, auxin transport, plant defense

## Abstract

Covalent attachment of the small modifier ubiquitin to Lys ε-amino groups of proteins is surprisingly diverse. Once attached to a substrate, ubiquitin is itself frequently modified by ubiquitin, to form chains. All seven Lys residues of ubiquitin, as well as its N-terminal Met, can be ubiquitylated, implying cellular occurrence of different ubiquitin chain types. The available data suggest that the synthesis, recognition, and hydrolysis of different chain types are precisely regulated. This remarkable extent of control underlies a versatile cellular response to substrate ubiquitylation. In this review, we focus on roles of Lys63-linked ubiquitin chains in plants. Despite limited available knowledge, several recent findings illustrate the importance of these chains as signaling components in plants.

Plant cells, similar to animal or fungal cells, have the ability to distinguish between substrates with single ubiquitin moieties attached, and between ubiquitin chains linked via different Lys residues. Mass spectrometric analysis of the *Arabidopsis* proteome showed that linkage of the ubiquitin carboxyl terminus to Lys 48 (K48) of ubiquitin most abundant, followed by K63 and by K11 linkages (Kim et al., 2013). **Figure 1** illustrates that different linkage types lead to differences in three-dimensional structure (for review, see also Kulathu and Komander, 2012). K48 and K11-linked ubiquitin chains result in proteolytic destruction of a substrate by the proteasome. In contrast, K63 chains are believed to have different consequences for the substrate. Although these latter chains can foster proteasomal degradation *in vitro* (Kirkpatrick et al., 2006), the *in vivo* significance of this finding remains to be demonstrated. A case in point is the mammalian “chain editing” enzyme A20, which removes K63 chains from its substrate RIP1 via a ubiquitin protease domain, to subsequently build a K48 chain via its ubiquitin ligase function. Whereas K63 chain-modified RIP1 is stable, the protein is quickly degraded after editing by A20 (Hymowitz and Wertz, 2010). It seems plausible that also in plants, the majority of K63 chains do not target substrates for proteasomal degradation. Plant enzymes and substrates for modification by K63 chains are largely unexplored, but first insights are emerging and, together with a glimpse at fungi and animals, shape expectations regarding conserved and plant-specific features.

**FIGURE 1:**
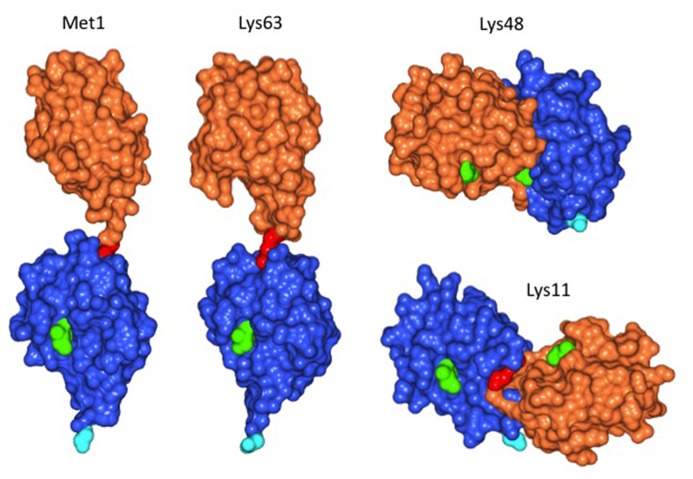
**Structure of diubiquitin linked via different Lys residues.** Linkage of donor ubiquitin (orange) to Lys63 of the acceptor ubiquitin (blue) results in positioning the donor opposite to the C-terminal Gly of the acceptor (cyan).This structure is similar, but not identical to ubiquitin linked via the amino-terminal Met, which is the co-translational peptide bond linkage of many ubiquitin precursors. In contrast, linkage of the donor to Lys48 gives a more compact structure, with the donor positioned on the side of the acceptor. Similarly, use of Lys11 for linkage positions the donor on the opposite side of the acceptor ubiquitin compared to Lys48 linkages. Longer chains of Lys48 or Lys11 linked ubiquitin are therefore more compact than Lys63 chains and are known as signals for proteasomal degradation, whereas Lys63 chains are believed to have distinct roles, independent of the proteasome. Amino acid Ile44 was colored in light green to highlight the orientation of ubiquitin moieties, the Lys residue of the acceptor and the C-terminal Gly of the donor used for linkage formation are colored in red. Images were withdrawn from RCSB protein data bank [2W9N (M1), 3H7P (K63), 2PE9 (K48), and 2MBO (K11)] and processed with program CCP4 Molecular Graphics.

Ubiquitin ligases that form K63 chains are not generally conserved across phyla. However, there exists at least one conserved component of K63 chain formation. The ubiquitin conjugating enzyme (Ubc)13 of *Saccharomyces cerevisiae*, together with a Ubc variant called methyl methane sulfonate sensitive (Mms)2, is dedicated to K63 chain formation and has homologs in all eukaryotes. Ubiquitin ligases that associate with this Ubc13/Mms2 heterodimer can form K63 chains. There is no exclusivity, though, because the same E3s may form different linkage types in association with other E2s, and mammalian and yeast E3s exist that form K63 chains without Ubc13/Mms2 participation. Based on interaction with an *Arabidopsis* homolog of Ubc13, a class of plant ubiquitin ligases termed RING DOMAIN LIGASE (RGLG) was identified that forms K63 chains *in vitro* (Yin et al., 2007). Additional candidates for K63 chain forming ligases are plant homologs of yeast Rad5 (Chen et al., 2008), although these enzymes remain to be characterized biochemically. It is possible that a requirement for K63 chains is conserved in certain biological processes, even though this conservation does not extend to the E2/E3 enzymes involved. As summarized in **Figure 2**, we discuss in the following examples of proven or suspected K63 chain requirement, most prominently DNA repair and membrane protein turnover, and additional publications pointing to roles for K63 chains in other processes.

**FIGURE 2:**
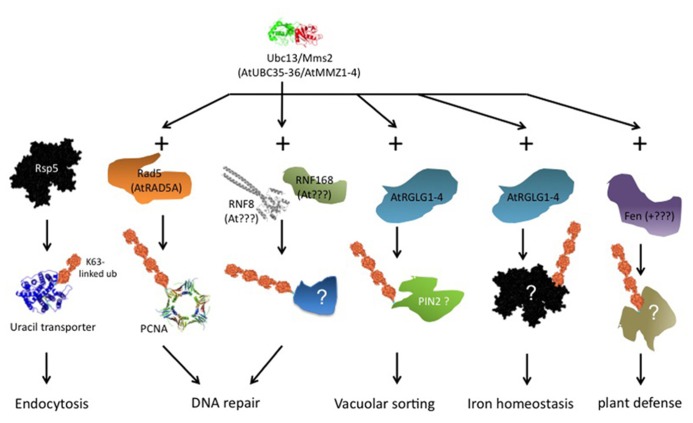
**Overview of ubiquitin K63 chain dependent processes.** The scheme is a composite of data from different organisms. The leftmost column, endocytosis of uracil permease Fur4 via ubiquitin ligase Rsp5, was investigated in *S. cerevisiae*. Ubiquitin transfer via Rsp5 occurs from a Cys residue in the ligase HECT domain, Ubc13/Mms2 is not involved. RNF8 and RNF168 (next columns) were characterized in mammals, and no plant homologs are known to date. The other schemes refer directly to processes in plants. See text for further details.

In animals, ubiquitin K63 chains have multiple roles in supporting DNA repair processes (for review, see Jackson and Durocher, 2013). One known contribution is the modification of DNA clamp protein proliferating cell nuclear antigen (PCNA) by a ubiquitin K63 chain during post-replication repair. If the DNA replication fork encounters an obstacle, ubiquitin ligase Rad18 together with E2 Rad6 modifies PCNA by addition of a single ubiquitin moiety, which leads to recruitment of an error-prone DNA polymerase for trans-lesion DNA synthesis. Alternatively, the single ubiquitin moiety is extended into a K63 chain by another ubiquitin ligase (called Rad5 in *S. cerevisiae*), together with the Ubc13/Mms2 heterodimeric E2. The chain fosters error-free repair by facilitating a template switch, so that damages blocking the replication fork can be bypassed using the sister strand as a template (Mailand et al., 2013). K63 chains are also essential for double strand break (DSB) repair. Two components of DSB repair, the RING finger domain containing ubiquitin ligases RNF8 and RNF168, associate with the Ubc13/Mms2 heterodimer to build K63 chains. RNF8 acts earlier by binding to phosphorylated histone H2AX, which is an early mark of broken DNA ends. K63 chain formation on as yet poorly defined substrates then allows recruitment of the next layer of the repair machinery, including RNF168, which contains ubiquitin-binding domains. K63 chain deposition by RNF168 is again necessary for recruitment of another set of repair factors. While the ubiquitin ligases RNF8 or RNF168 have no reported plant homologs, Ubc13 and Mms2 homologs of *Arabidopsis* can complement the DNA repair defects of the respective yeast mutants (Wen et al., 2006, 2008). More importantly, *Arabidopsis* mutants in one Mms2 homolog exhibit increased sensitivity to a DNA damaging agent (Wen et al., 2008). Likewise, mutants in an *Arabidopsis* homolog of ubiquitin ligase Rad5 lead to DNA damage sensitivity (Mannuss et al., 2010; Wang et al., 2011), and support a role for template switching in plant post-replication repair (Mannuss et al., 2010). These studies are also consistent with a role for plant RAD5 in DNA repair outside the S-phase (suggesting modification of additional substrates besides PCNA).

Attachment of one ubiquitin moiety to membrane proteins is part of the endocytosis process, and several components of endosomal sorting complexes required for transport (ESCRT complexes) have ubiquitin binding domains (Korbei and Luschnig, 2013). The ubiquitylation of membrane receptors typically occurs after ligand binding. For unclear reasons, some membrane proteins require not only monoubiquitin modification for internalization, but ubiquitin K63 chain attachment. The chain may be short, as shown for the yeast uracil transporter Fur4 (Lauwers et al., 2010), but this short ubiquitin K63 chain is nonetheless essential for endocytosis. The simplest explanation for the requirement is a geometric necessity: if single ubiquitin moieties would be sterically not accessible for ESCRT factors, short ubiquitin K63 chains could act as “honorary monoubiquitin” modifications, promoting substrate endocytosis.

Once a membrane protein is sequestered into a vesicle, productive association with other proteins can continue. For instance, signaling may occur in different or unaltered form compared to the plasma membrane-localized receptor (Jalink and Moolenaar, 2010), and, more important in this context, the role of ubiquitin is not finished. There are many indications that later steps of membrane trafficking require ubiquitylation, and again K63 chains are suspected to play an important part. One of the decisions to be made while a membrane protein is on a vesicle is whether it is re-cycled onto the plasma membrane, or whether it is diverted into the vacuole via multivesicular bodies. In that case, ubiquitin K63 chains can effect proteolytic destruction of substrates, which occurs via vacuolar degradation, rather than by the proteasome.

In mammals, K63 ubiquitin chain-specific enzymes exist that critically influence the vacuolar (lysosomal) versus plasma membrane path of endocytosed membrane proteins (Clague and Urbe, 2010; Wright et al., 2011). Proteins with homology to ubiquitin K63 chain-specific proteases of mammals were investigated in *Arabidopsis* (Isono et al., 2010; Katsiarimpa et al., 2011). Although the plant proteases are not specific for K63 chains, but can also hydrolyze K48 chains, they have a role in vacuolar sorting, just as the mammalian counterparts.

A direct link between ubiquitin K63 chains and plant plasma membrane proteins was established when analyzing turnover of the PIN2 auxin efflux facilitator. Here it turned out that PIN2 decoration by K63 chains functions in efficient endocytic sorting to the vacuole (Leitner et al., 2012). Notably, when testing a *pin2* allele mimicking constitutive mono-ubiquitylation, but lacking further lysines required for its K63-linked ubiquitylation, the fusion protein got stuck *en route* to the vacuole, highlighting an essential function for K63 chains at later steps of PIN2 endocytic sorting. Analysis of PIN2 in an *rglg* mutant combination deficient in K63 chain-forming E3 ligases revealed reduced ubiquitylation levels, which establishes PIN2 as potential RGLG substrate, but direct evidence for such interaction remains to be shown (Yin et al., 2007; Leitner et al., 2012).

An interesting connection was found between ubiquitin K63 chains and iron homeostasis (Li and Schmidt, 2010). Iron deficiency leads to the up-regulation of the Ubc13 homolog in cucumber, which occurs on the protein level without increase of the transcription rate. In line with a role for K63 chains in iron response, a mutation in one of the *Arabidopsis* Ubc13 homologs results in decreased formation of branched root hairs, which are normally formed in response to iron deficiency. Mutations in both Ubc13 homologs lead to a generally decreased density of root hairs. Li and Schmidt (2010) also investigated iron responses in a mutant in two K63 forming ubiquitin ligases, RGLG1 and RGLG2. The double mutant displays, unexpectedly, constitutive formation of forked root hairs, suggesting the existence of additional players in this regulon with impact on K63 chains. For example, this response could involve variations in the abundance of iron transport proteins such as IRON-REGULATED TRANSPORTER1 (IRT1), a key effector of iron availability in *Arabidopsis* (Vert et al., 2002). Ubiquitylation and associated vacuolar targeting of IRT1 has been demonstrated recently, and another E3 ligase termed IRT1 DEGRADATION FACTOR1 (IDF1) has been implicated in such regulation (Barberon et al., 2011; Shin et al., 2013). However, IRT1 appears to be mono-ubiquitylated at one or several sites, whereas modification by K63 chains in a dynamic control of IRT1 turnover has not been demonstrated yet (Barberon et al., 2011). It thus remains unclear whether or not K63 chains could influence iron homeostasis at the level of carrier turnover.

Ubiquitin K63 chains are also important for cytosolic signaling complexes assembled as part of defense responses. Mural et al. (2013) identified Fen-interacting-protein (Fni)3, a tomato Ubc13 homolog, and its cofactor *Solanum lycopersicum* Ubc variant (Suv; an Mms2 homolog) as mediators of the defense response. Fen is a protein kinase involved in recognition of pathogen effector proteins, representing truncated variants of the bacterial E3 ligase AvrPtoB (Abramovitch et al., 2003), and Fen interaction with Fni3 has been suggested to affect Fen-mediated signaling to promote cell death upon *Pseudomonas* infection (Mural et al., 2013). E3 ligases and potential substrates for Fni3/Suv-mediated K63 chain formation remain to be identified. It should be noted that the *Arabidopsis* plasma membrane-localized flagellin receptor FLS2, and probably other pathogen-associated molecular pattern (PAMP) receptors, represent likely substrate for AvrPtoB-mediated ubiquitylation and degradation (Göhre et al., 2008). It will be interesting to learn if and how all these pathways interact, and how ubiquitin K63 chain formation controls immune responses in higher plants.

Taken together, current knowledge concerning ubiquitin K63 chains in plants is sufficient to recognize their importance, but not yet sufficient to allow, in any individual case, a mechanistic description of how K63 chains are integrated into biological processes. Future research thus holds promise for exciting insights into this topic.

## Conflict of Interest Statement

The authors declare that the research was conducted in the absence of any commercial or financial relationships that could be construed as a potential conflict of interest.
